# A randomized double-blind cross-over trial to study the effects of resistant starch prebiotic in chronic kidney disease (ReSPECKD)

**DOI:** 10.1186/s13063-022-06009-1

**Published:** 2022-01-24

**Authors:** Maryam Shamloo, Rebecca Mollard, Haizhou Wang, Kulwant Kingra, Navdeep Tangri, Dylan MacKay

**Affiliations:** 1 Chronic Disease Innovation Centre, Seven Oaks General Hospital, Winnipeg, MB Canada; 2 Department of Human Nutritional Sciences, University of Manitoba, Winnipeg, MB Canada; 3 Department of Community Health Sciences, University of Manitoba, Winnipeg, MB Canada; 4 Max Rady College of Medicine, University of Manitoba, Winnipeg, MB Canada

**Keywords:** Chronic kidney disease, Resistant starch, Gut microbiome, Uremic toxins, Randomized controlled trial

## Abstract

**Background:**

Chronic kidney disease (CKD) is associated with a reduced quality of life and an increased risk of kidney failure, cardiovascular events, and all-cause mortality. Accumulation of nitrogen-based uremic toxins leads to worsening of symptoms in individuals with CKD. Many uremic toxins, such as indoxyl and p-cresol sulphate, are produced exclusively by the gut microbiome through the proteolytic digestion of aromatic amino acids. Strategies to reduce the production of these toxins by the gut microbiome in individuals with CKD may lessen symptom burden and delay the onset of dialysis. One such strategy is to change the overall metabolism of the gut microbiome so that less uremic toxins are produced. This can be accomplished by manipulating the energy source available to the microbiome. Fermentable carbohydrates which reach the gut microbiome, like resistant starch (RS), have been shown to inhibit or reduce bacterial amino acid metabolism. This study aims to investigate the effects of resistant potato starch (RPS) as a prebiotic in individuals with CKD before the onset of dialysis.

**Methods:**

This is a double-blind, randomized two-period crossover trial. Thirty-six eligible participants will consent to follow a 26-week study regimen. Participants will receive 2 sachets per day containing either 15 g of RPS (MSPrebiotic, resistant potato starch treatment) or 15 g cornstarch (Amioca TF, digestible starch control). Changes in blood uremic toxins will be investigated as the primary outcome. Secondary outcomes include the effect of RPS consumption on symptoms, quality of life and abundance, and diversity and functionality of the gut microbiome.

**Discussion:**

This randomized trial will provide further insight into whether the consumption of RPS as a prebiotic will reduce uremic toxins and symptoms in individuals who have CKD.

**Trial registration:**

ClinicalTrials.govNCT04961164. Registered on 14 July 2021

## Background

In Canada, the overall prevalence of chronic kidney disease (CKD) is ~ 13%, rising to ~ 30% in those over 65 [1]. CKD is associated with a reduced quality of life and an increased risk of kidney failure, cardiovascular events, and all-cause mortality [2–4]. An ageing population along with increasing rates of hypertension and diabetes mellitus are among the factors related to an increasing prevalence of end-stage kidney disease [5]. As kidney function declines, there is an accumulation of nitrogen-based uremic toxins which leads to worsening of symptoms and complications. Many uremic toxins, such as indoxyl and p-cresol sulphate, are produced exclusively by the gut microbiome [6] through the proteolytic digestion of aromatic amino acids (tyrosine and tryptophan, respectively) [7]. Strategies to reduce the concentration of these toxins present a low-risk, low-cost opportunity to lessen symptom burden in patients with CKD and may delay the onset of dialysis.

Indoxyl and p-cresol sulphate are thought to have negative effects on multiple organ systems and have been associated with reported clinical symptoms, such as uremic pruritus [8], and cardiovascular mortality in individuals with CKD [9–13]. Two approaches have been identified to deal with these toxins: binding or removing the toxins in the gut, or reducing their production [11]. Recent attempts to pharmacologically bind uremic toxins in the gut with activated charcoal have been unsuccessful in clinical trials [12], perhaps because the binding was not effective, or because the burden on participants (30 pills per day) was too much to maintain compliance. Strategies to reduce the production of these toxins by the gut microbiome in individuals with CKD may be more effective, especially if they have a lower patient burden [14]. One such strategy is to change the overall metabolism of the gut microbiome so that less uremic toxins are produced. This can be accomplished by manipulating the energy source available to the microbiome [7, 14]. Fermentable carbohydrates that reach the gut microbiome, like resistant starch, have been shown to inhibit or reduce bacterial amino acid metabolism [15, 16]. Raw potato starch (RPS), which is a R2-resistant starch, has been shown to increase carbohydrate-degrading bacteria, such as Bifidobacteria, and decrease bacteria with proteolytic activity, such as *Escherichia coli* [14, 17, 18]. RPS has also been shown to reduce the concentrations of gut microbiome-derived uremic toxins in pigs [19].

While animal and human studies involving resistant starch have shown the ability to change the gut microbiota and reduce the amount of uremic toxins, there are limited studies in individuals with CKD [14]. However, in one study conducted in individuals with CKD on dialysis, a reduction in uremic toxins following high-amylose cornstarch consumption for 6 weeks was observed [20]. High-amylose cornstarch is ~ 60% resistant starch by dry weight, whereas the RPS to be used in this proposal is ~ 70% resistant starch and ~ 10% other dietary fibres [17].

To our knowledge, no studies have investigated the effects of RPS as a prebiotic in individuals with CKD before the onset of dialysis.

## Methods/design

### Study design

**Fig. 1:**
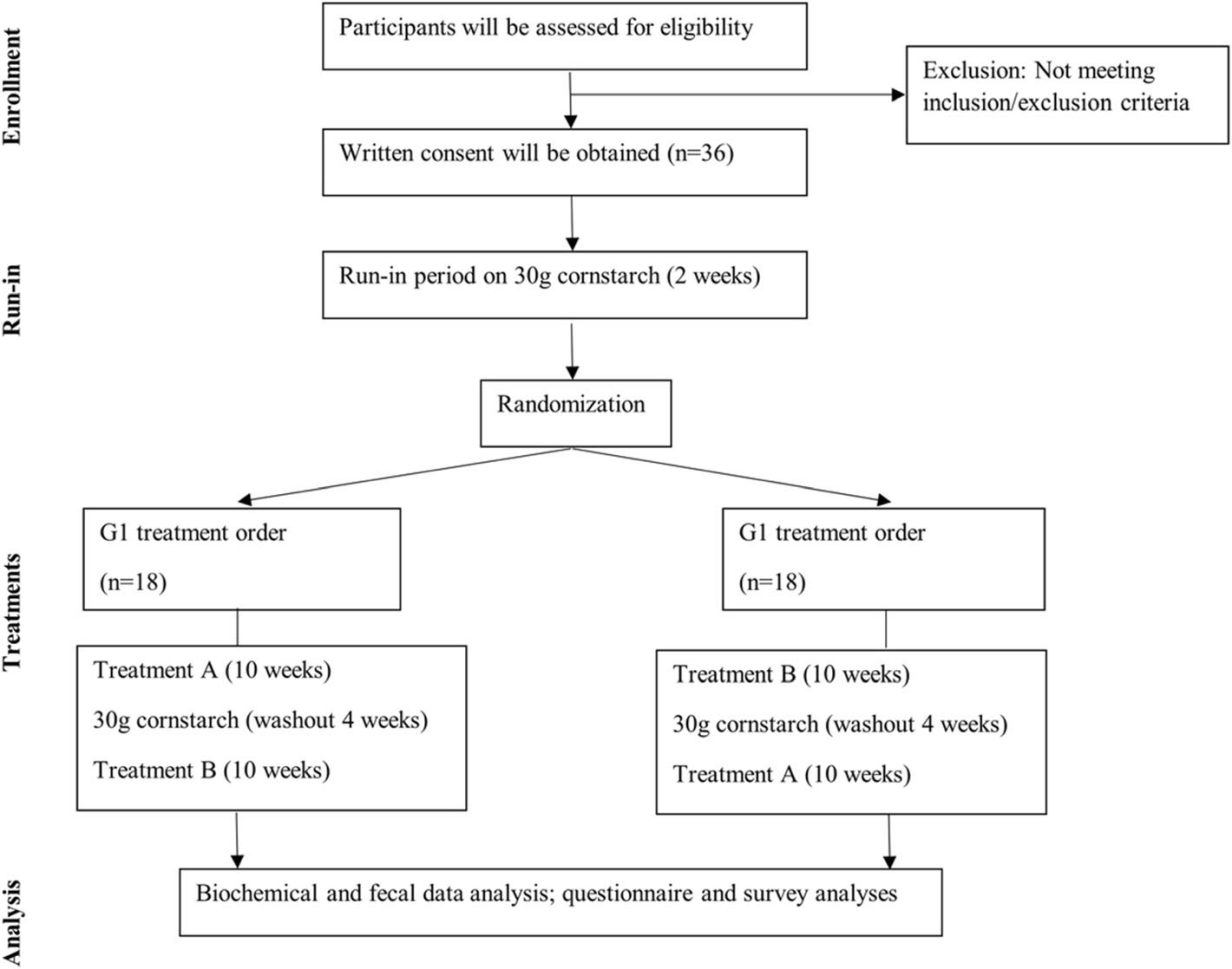
Study protocol flow chart

The clinical trial is exploratory and will follow a 2-period double-blind cross-over design. The allocation ratio will be 1:1. It will take place at the Chronic Disease Innovation Centre (CDIC) at Seven Oaks General Hospital in Winnipeg, Canada. The study protocol flow chart is shown in Fig. 1. This trial will be conducted in compliance with Good Clinical Practice (GCP) and all local and national guidelines.

Participants will consent to follow a 26-week study regimen. Each participant will receive 2 sachets per day containing either 15 g of RPS (MSPrebiotic, resistant potato starch treatment) or 15 g cornstarch (Amioca TF, digestible starch control). The powder in the sachets will be mixed in water and consumed, one sachet in the morning and one before bed. Participants will be instructed to consume the investigational product at least 2 h prior to or after taking any medication.

For the first 2 weeks, participants will go through a run-in period, where they will all receive the cornstarch control. During weeks 3 to 12 (period 1), participants will receive either RPS or cornstarch. The first treatment received will be determined by a randomization procedure. During weeks 13 to 16, participants will undergo a washout period where they will all consume cornstarch. During weeks 17 and 26 (period 2), participants will receive the treatment they did not previously consume (RPS or cornstarch, respectively).

Participants will complete questionnaires and food records at the beginning and end of each treatment period (Table 1). They will also provide blood, urine, and faecal samples for analysis (Table 1).

**Table 1:** The schedule of enrolment, interventions, and assessments

Assessment	Screening and enrolment	Visit 1	Visit 2	Visit 3	Washout	Visit 4	Visit 5
Periods		Run-in		Period 1		Period 2	
Day in study		1	15	84	85–112	113	182
Informed consent	□						
Screening	□						
Demographics	□						
Medical history anthropometrics	□	□	□	□		□	□
Dietary recalls			□	□		□	□
SF-36, ESAS			□	□		□	□
Blood, urine, and stool collections			□	□		□	□
Adverse event			□	□		□	□

### Study participants

Thirty-six adults (18–85 years) with CKD will be recruited to the study via the advanced CKD clinic at Seven Oaks Hospital. Dr. Tangri and Dr. Mollard will oversee the recruitment.

#### Inclusion criteria

The participant may enter the trial if all of the following apply: the participant is willing and able to give informed consent for participation in the trial and has the ability to speak and read English; male or female, aged 18 years or above; females of child-bearing potential must agree to use a medically approved method of birth control for the duration of the study; all hormonal birth control must have been in use for a minimum of 3 months; acceptable methods of birth control include hormonal contraceptives including oral contraceptives, hormone birth control patch, vaginal contraceptive ring, injectable contraceptives, hormone implant, double-barrier method, intrauterine devices, and non-heterosexual lifestyle or agrees to use contraception if planning on changing to heterosexual partner(s) and vasectomy of partner at least 6 months prior to screening; the estimated glomerular filtration rate (eGFR) is < 15 mL/min/1.73 m^2^ for the past 3 months; and in the investigator’s opinion, participants are able and willing to comply with all the trial requirements.

#### Exclusion criteria

The participant may not enter the trial if any of the following apply: the participant is cognitively impaired and cannot give consent or participate in the group programme; the participant has an existing relationship with the research team, such as supervisory relationship (student, employee) or familial relationship (child, spouse, etc.); participants who indicate that they cannot consume study treatments; participants who indicate they are allergic to potatoes or corn; female participants who are pregnant, lactating, or planning pregnancy during the trial; history of renal transplant, ongoing dialysis, use of antibiotics (last 3 months), bowel diseases, cancer, surgically removed bowel, or any gastrointestinal surgery (e.g. intestinal resection, gastric bypass, colorectal surgery); inability to consume treatment due to swallowing or GI issues; participating in another interventional trial that could influence the intervention or outcome of this trial; participants with uncontrolled diabetes with A1C > 10%; participants who consume probiotic supplements; participants with abnormal constrictions of the gastrointestinal tract, diseases of the oesophagus, and/or the superior opening of the stomach (cardia), potential or existing intestinal blockage, paralysis of the intestine, megacolon, faecal impaction, appendicitis, a sudden change in bowel habits that has persisted for more than 2 weeks, undiagnosed rectal bleeding, or failure to defaecate following the use of another laxative product; participants with severe anaemia (haemoglobin less than 70); participants taking medications which inhibit peristaltic movement (e.g. opioids, loperamide); and participants able to maintain high fibre/adequate fibre intake through diet or taking other fibre supplements.

#### Recruitment

A research coordinator will work with someone within the circle of care (such as a nurse, a clinical clerk, or a physician) of patients attending the Manitoba renal programmes interdisciplinary CKD clinic at Seven Oaks Hospital to pre-identify potentially eligible participants.

The first point of contact will occur during routine clinical care or through virtual meetings. An individual in the patient’s circle of care will inform the patient that they may be eligible to participate in a research study and ask for their permission for the research coordinator to contact them to discuss the study in detail that day or at a later time, through a virtual or in-person visit. This is meant to be a brief conversation, and the main purpose is to seek permission for the individual to be contacted by the research coordinator and to do this via someone in the patient’s circle of care. We will ensure that all clinical conversations (e.g. patient’s health, updates, appointment reminders) will be completed first before the study is introduced to them.

The second point of contact will be made by the research coordinator through virtual meetings or in-person visits to explain the study in greater detail to the patient. Patients that are interested in the study will be given a letter of information and consent form to review and sign through the Research Electronic Data Capture (REDCap) platform or in paper form. A participant ID log will be kept recording the patients who enrol into the study. It is expected that the recruitment of the 36 participants will occur over a 3-month period.

### Treatments

The treatments will consist of (1) 30 g of RPS (MSPrebiotic) or (2) 30 g digestible cornstarch (Amioca TF) per day. Digestible cornstarch was chosen as the comparator because it is visually similar to the RPS but contains no resistant starch. These treatments will be given to participants in 2 sachets per day, which are to be mixed with water and consumed, one in the morning and one in the evening before bed. The dosage of 30 g is based on a previous study of RPS in older adults [17]. The resistant potato starch and cornstarch will be pre-packed in labelled packages provided by the manufacturer. The cornstarch and resistant potato starch sachets will be stored at room temperature until it is dispensed to participants. Courier will be available as an option to deliver the packages to the study participants.

### Compliance

Diaries with daily checklists for the 2 sachets will be given to the participants for each day during the run-in, washout, and treatment periods. These will be used to monitor their compliance with the study protocol. Additionally, participants will be instructed to return all empty sachets for counting purposes. Compliance for both groups will be defined as an attendance of ≥ 90% of the scheduled visits with the research team and ≥ 80% of the required treatments consumed according to diary entries and returned sachets. Participants will be monitored every week by the research team through phone calls or online videos. Additionally, there will be a study dispensing log which will be used to track all study treatments given to each of the participants. This log will include participant ID, date of dispensing, randomization code, and the returned sachet counts. Concomitant medication will be recorded at the end of the run-in and each treatment period.

### Assessments

#### Baseline assessments

After screening, eligible participants will receive cornstarch sachets for the first 2 weeks during a run-in phase; however, they will be instructed that they could be receiving cornstarch or resistant potato starch. Besides cornstarch sachets, participants will also receive the Medical Outcomes Study Short Form 36-item (SF-36) and Edmonton Symptom Assessment Scale (ESAS) questionnaires, the link to an Automated Self-Administered 24-h Canada (ASA24) dietary recall survey, a tape measure, a weight scale, and faecal sample collection kits.

At the end of the second week of the run-in period, study coordinators will book a virtual/in-person meeting with participants to instruct and measure body weight and waist circumference, go through medication/supplement condition, and give a general instruction on how to fill in SF-36, ESAS, and ASA24 questionnaires. Study coordinators will also give a brief introduction about blood, urine, and faecal collection steps and then send a lab requisition to the participants.

#### Ongoing assessments

Participants will be able to contact the study team at any time and will be given options to do so (text, voice call, video link, urgent in-person appointment). After enrolment in the study, participants will have follow-up virtual/in-person study visits as described in Table 1. Study visits will be acceptable on days within ± 5 days.

### Outcome measures

Serum concentrations of indoxyl and p-cresol sulphate are considered the primary outcome of this trial. This is to investigate how RPS consumption influences blood uremic toxins. The abundance, diversity, and functionality of the gut microbiome will be investigated as the secondary outcomes. Samples will be sent out for blood, faecal, and urine metabolomics analysis and gut microbiome analysis, following shipping guidelines of the contracted service providers, University of Manitoba policies, and all applicable local and national regulations.

#### Uremic toxin measurement

Serum samples of indoxyl and p-cresol sulphate will be collected by the start and end of each treatment period. The concentrations of free and total p-cresol sulphate and indoxyl sulphate will be measured by high-pressure liquid chromatography [21]. Total p-cresol sulphate and indoxyl sulphate will be measured after deproteinization of serum with ethanol. These analyses will be conducted at McMaster University in Hamilton, ON.

#### Gut microbiome

Two faecal samples will be collected from consecutive days for analysis at the end of the run-in and each treatment period. Participants will collect the faecal sample; they will be provided collection kits and an ice pack and instructed to collect a single sample from 3 separate places on the stool using a spoon attached to the cap of the collection tube. Participants will be instructed to store the collected faecal samples in their household − 20 °C freezer with the provided ice pack, until transport back to the study centre. At the study centre, samples will be aliquoted and then stored at − 80 °C. Faecal samples will undergo genomic DNA extraction following the manufacturer’s protocol. Experimental negative controls will be included in extraction protocols to confirm the reliability and consistency of the extracted nucleic acid. The V4 hypervariable region of 16S rRNA gene will be amplified; the sequencing library will be generated and sequenced by Microbiome Insights (www.microbiomeinsights.com) in Vancouver, BC.

#### Anthropometry

Body weight and waist circumference will be measured at the beginning and end of each period.

#### Edmonton Symptom Assessment Scale (ESAS)

ESAS will be completed at the beginning and end of each period through paper or online by REDCap. The ESAS is a widely used tool for measuring physical and psychological symptom distress that has been validated in individuals with CKD [22], which consists of nine visual analogue scales (0–10 scale) for pain, activity, nausea, depression, anxiety, drowsiness, appetite, well-being, and shortness of breath. The scale for each symptom is anchored by the words “no” and “severe” at 0 and 10, respectively.

#### Quality of life

The Medical Outcomes Study Short Form 36-item Questionnaire (SF-36) will be used to measure the quality of life and has been validated in individuals with CKD [23]. SF-36 will be filled in by the beginning and end of each period. The questionnaire is designed for use across diverse populations and healthcare settings and is composed of eight scales: physical functioning (PF), role functioning/physical (RP), bodily pain (BP), general health (GH), vitality (VT), social functioning (SF), role functioning/emotional (RE), and mental health (MH). These scales are scored from 0 to 100, with higher scores indicating better function.

#### Dietary intakes and nutritional assessment

Participants will complete three dietary recall surveys (for two weekdays and one weekend) following the run-in, and in the last week of each treatment period, using the Automated Self-Administered 24-h Canada (ASA24®) dietary assessment tool. The ASA-24 is a web-based tool that enables multiple, automatically coded, self-administered 24-h recalls. Participants will receive training and have support from study staff in filling out the recalls.

#### Clinical chemistry

Blood and urine samples for clinical chemistry will be collected at the start and end of each treatment period. In the blood, albumin, BUN, bicarbonate, calcium, chloride, creatinine, eGFR, glucose, phosphorus, potassium, sodium, and HbA1c will be measured. In the urine, albumin, albumin/creatinine ratio, glucose, and total protein will be measured. All samples will be collected, and analytes will be measured by Shared Health Diagnostics at Seven Oaks Hospital.

#### Metabolomics

Serum, faecal, and urine samples will be collected at the end of run-in and treatment periods. The metabolomics analyses and informatics will be conducted by McMaster University in Hamilton, ON. Targeted analysis of water-soluble metabolite classes including amino acids, sugars, alcohols, organic acids, amines, TCA cycle intermediates, and short-chain fatty acids using quantitative NMR spectroscopy will be conducted on serum collected at the endpoint of each study period [24]. A targeted quantitative metabolomics approach will be used to analyse urine samples using a direct injection mass spectrometry with a reverse-phase LC-MS/MS assay. Serum and urine samples will be collected in accordance with metabolomic sample collection guidelines, allowing as much as possible that the original metabolic profile of the fresh samples is maintained and minimizing potential pre-analysis sample collection or handling issues that could bias the results of the metabolomic analyses [25].

### Sample handling

Blood and urine samples will be collected by the certified phlebotomist in Shared Health Diagnostics at Seven Oaks Hospital. The laboratory will process and run the analysis for the clinical chemistry and store the serum and urine sample in − 80 °C freezer in the Shared Health lab for uremic toxic measurement and metabolomics analysis. The blood and urine samples will be collected by the start and end of each treatment phase. For stool samples, participants will be provided collection kits and an ice pack and instructed to collect a single sample from 3 separate places on the stool using a spoon attached to the cap of the collection tube. Participants will be instructed to store the collected faecal samples in their household − 20 °C freezer with the provided ice pack, until transport back to the study centre. At the study centre, samples will be aliquoted and then stored at − 80 °C in the Shared Health Diagnostics Lab. These samples may be moved to storage located at the University of Manitoba if required due to space constraints. Faecal samples will be collected at the beginning and end of treatment periods 1 and 2. At least one sample will be obtained during those two consecutive days if possible. Samples will be sent out for blood, faecal, and urine metabolomics analysis and gut microbiome analysis, following shipping guidelines of the contracted service providers, University of Manitoba policies, and all applicable local and national regulations.

### Qualified investigator responsibilities

The qualified investigator (QI) will be responsible for determining the eligibility of individuals to participate in the trial. Although some tasks may be delegated to other qualified clinical trial staff members, the QI will ensure the individual or party is qualified to perform those study tasks and is responsible for supervising any individual or party to whom tasks are delegated at the trial site. The QI assumes ultimate responsibility for the overall conduct of a clinical trial and for compliance with all applicable regulations and guidelines. The QI must document the delegation of tasks/duties. The QI will evaluate the general health and review the participants’ medical history, vital sign results, concomitant medications, and all laboratory results to determine the eligibility for the trial. The QI will have ongoing responsibilities of monitoring adverse events, serious adverse events, concomitant medications, and any additional laboratory results to ensure participant safety through the entire clinical trial. All study-related medical decisions are made, unless delegated to a qualified and trained sub-investigator by the QI. The QI is ultimately responsible for the welfare and safety of all participants on the trial. The QI agrees that the site will permit, if required, study-related monitoring, audits, REB review, and regulatory inspection(s), providing direct access to source data/documents. It is the responsibility of the QI or designee to maintain adequate clinical study records. Copies of all clinical study material must be archived for a period defined by Canadian regulations. It is also the responsibility of the QI to ensure that the study is conducted in accordance with the principles of Good Clinical Practice and according to the applicable local laws and regulations concerning studies conducted on human participants which are outside the definition of a medical product or medical device. The QI will have access to all product codes and may break randomization codes if necessary, for handling an adverse event. Lastly, the QI or their designee will review the deviations and determine if the deviation(s) would significantly affect the results and, if deemed necessary, not include such data in statistical analysis. The QI will ensure that all study documents are maintained up to date as specified in the Essential Documents for the Conduct of a Clinical Study and as required by the applicable regulatory requirements. The QI will ensure that all serious adverse events are immediately reported to Health Canada, the University of Manitoba REB, and any other applicable regulatory authorities.

### Safety monitoring committee

The interventions in this trial are low risk, and they are in addition to standard care that the participants will continue to receive throughout the trial period and after the trial is completed; therefore, no data and safety monitoring board (DSMB) will be formed. However, there will be an external review of the participant clinical chemistry and any trial AEs at the trial midpoint when all participants have completed their first treatment period. This review will be conducted by a nephrologist from outside of Manitoba. This review will monitor evidence for treatment harm, looking for trends in the clinical chemistry and/or increases in un/expected events, related to the treatments and take appropriate action. These actions may include proposing protocol changes they could include early stopping of the trial due to clear harm of a treatment depending on the results of the review.

### Statistical analysis

The bioinformatics and statistical analyses of microbiome data will be performed with the assistance of Microbiome Insights and the Data Science platform at CHI; it will be updated based on the recommendations and technology advancements between now and the processing of the samples. In general, the default settings of the FLASH assembler [22] will be used to merge the overlapping paired-end Illumina fastq files. UPARSE algorithm [23] will be used for (a) quality filtering of the reads based on the maximum expected error value = 1.0, (b) identification of unique sequences, (c) abundance sorting and removal of singletons, (d) clustering the reads into operational taxonomic units (OTUs) based on 97% identity threshold, (e) de novo and reference-based chimera checking (against GOLD database [22]), and (f) construction of OTU table. Taxonomic classification will be then carried out using QIIME [26] implementation of UCLUST [24] and will be aligned against the Greengenes database using the PyNAST algorithm [25]. Phylogenetic trees were built with FastTree [27] for further comparison between microbial communities. Prior to performing downstream analyses, the resulting OTU table will be filtered to remove all the samples with low-sequencing depths. Community richness and diversity indices will be then calculated using QIIME at a given even depth per sample. Phylogenetic- (weighted UniFrac distances) and abundance-based (Bray-Curtis dissimilarity) β-diversity metrics will be calculated following normalization of the final OTU table using the cumulative sum scaling (CSS) transformation [28]. Principal coordinate analysis (PCoA) will be applied on the resulting distance matrices to generate two-dimensional plots using default settings of the PRIMER-6 software (PRIMER-E Ltd, Plymouth). Unsupervised clustering analysis will be performed to relate clustering patterns of samples to the proportion of the core OTUs within each niche (core OTUs defined as those present in at least 75% of samples in each niche). The relative abundances of the selected OTUs will be normalized across samples. Bray-Curtis dissimilarities will be calculated using the R “vegan” package [29], and the resulting matrix will be subjected to unsupervised hierarchical clustering using the R “dendextend” package [30] and will be visualized over the heatmap of abundance matrix using the R “complexheatmap” package [31]. The UNIVARIATE procedure of SAS will be used for testing the normality of residuals for α-diversity measurements. Non-normally distributed data will be either log or Box-Cox power transformed and then subjected to analysis of variance (ANOVA) test using MIXED procedure of SAS. All pairwise comparisons among the groups will be tested using Tukey studentized range adjustment. Permutational multivariate analysis of variance (PERMANOVA; implemented in the Primer6 software) will be used to detect significant differences between β-diversity metrics of microbial communities. The relative abundances of selected core OTUs will be tested for statistically significant associations with available metadata using multivariate analysis with linear modelling (MaAsLin) [32] accounting for all potential confounders (covariates) that could be associated with the profile of microbiome (i.e. sex, age, BMI) and participants (treated as a random factor). Significant associations will be considered below a *q*-value threshold of 0.1. To assess the shifts in functionality of microbiome, correlation network analysis (CoNet, [33]) will be used to explore microbial co-occurrence/mutual-exclusion relationships and identify hub OTUs that show the highest number of positive/negative correlations with other OTUs under treatment conditions.

Effects of treatment on linear outcomes at the end of each period will be analysed by the SAS MIXED (SAS 9.4) procedure. Sequence and sex will be included in the model as fixed factors, and participants will be included as a repeated factor. The normality of the data distribution will be done using the Shapiro-Wilk test, and the non-normal variables will be transformed prior to analysis. Demographic data will be reported as the mean ± standard deviation. The results will be reported as least-squared means ± standard error of the mean (SEM) unless otherwise specified. Statistical significance will be set at *p* < 0.05 for all analyses. The Data Science Platform in CHI will provide data management support in addition to biostatistics support for the project.

### Randomization, blinding, and code-breaking

Eligible patients (*n* = 36) will go through assessments at baseline and will be randomly allocated to 2 groups, each consisting of 18 participants. Randomization will be done by an independent researcher in the biostatistics platform at the George and Fay Yee Centre for Healthcare Innovation (CHI) at the University of Manitoba. Randomization will be performed using code written in the R statistical programming language (version 3.5.3). Treatments will be assigned with a 1:1 ratio. A total of 48 randomization cards will be prepared, one set of 24 for each sex. The randomization schedule will be transferred into sets of opaque sealed envelopes. After a participant’s baseline visit, the study staff will open a sealed envelope which will contain the participant’s allocation. The order of the interventions will be blinded for both the investigators and the participants. Treatments will be given in sealed sachets; sachet content will be blinded by an outside party, given to the study staff labelled as A or B, and dispensed to participants according to their treatment period. Treatments will not be unblinded until the analyses are complete, unless required due to adverse events during the clinical trial. There will also be a midpoint review of the participants’ clinical data by an independent nephrologist who will be unblinded (see the “Qualified investigator responsibilities” section).

### Sample size calculation

A final samples size of 30 participants in this study will be able to detect a difference between the treatments in total p-cresol sulphate of 17.5 μmol/L, or ~ 15% change, at a power of .88 (alpha = 0.05, two-sided), given a within-person correlation of .79 [23] and an estimated standard deviation of 45 μmol/L [9] for total p-cresol sulphate. A 30% or greater drop in uremic solutes would be considered clinically significant, warranting additional trials investigating this prebiotic intervention in CKD, and we are confident we would be able to detect such a change should it occur. To account for the loss of power due to dropouts, we will recruit 36 participants.

#### Inclusion in analysis

The primary analysis will be conducted using the All Participants (intent-to-treat) analysis set. The primary analysis will be repeated in the *Completers* analysis set. Demographics and all other baseline measurements will be analysed in the All Participants set as well as in the Completers set.

Completers analysis set: all participants who have completed the trial

All Participants analysis set: all randomized participants

### Discontinuation/withdrawal of participants from trial treatment

Each participant has the right to withdraw from the trial at any time. Participants may discontinue trial participation at any time and are requested to contact a research team member to inform them of their decision. In addition, the investigators may discontinue a participant from the trial at any time if the investigators consider it necessary for any reason including pregnancy, ineligibility (either arising during the trial or retrospectively having been overlooked at screening), significant protocol deviation, significant non-compliance with the protocol, disease progression which results in the inability to continue to comply with the protocol, withdrawal of consent, and loss to follow-up.

Withdrawal will not result in the exclusion of the data for that participant from the analysis. As the primary analysis will be based on an intention-to-treat, there will also be a completer only analysis performed.

If a participant is withdrawn within the first 4 weeks of the trial, they will be replaced. If the replacement participant is withdrawn, there will be no subsequent replacement.

The reason for withdrawal will be recorded in the CRF, if provided.

### Remuneration

Participants will be remunerated $200 for each completed period or the prorated value if they withdraw from the trial. Each participant would receive $400 in total if they complete the full trial.

Participant’s name and address will be used for preparing, printing, mailing, and financial record keeping or remuneration cheques. Pre-paid postage and envelope will be provided to the participant with a form requiring their signature upon receipt of the remuneration cheque. Participants will be asked to return the form to CDIC. This record will be kept for a maximum of 7 years.

## Discussion

CKD has been associated with changes in gut microbial ecology, or “dysbiosis”, which may contribute to disease progression. Individuals and animals with CKD exhibit profound alterations in the gut environment including shifts in microbial composition, increased faecal pH, and increased blood levels of gut microbe-derived metabolites. Recent studies have focused on dietary approaches to favourably alter the composition of the gut microbial communities as a treatment method in CKD. Resistant starch (RS), a prebiotic that promotes the proliferation of gut bacteria such as *Bifidobacteria* and *Lactobacilli*, increases the production of metabolites including short-chain fatty acids, which confer a number of health-promoting benefits. Studies from animal models and individuals with CKD show that RS supplementation attenuates the concentrations of uremic retention solutes, including indoxyl sulphate and p-cresol sulphate. The fermentable dietary fibre-high amylose maize-resistant starch type 2 (HAMRS2) has been shown to alter the gut milieu in CKD rat models leading to markedly improved kidney function. RPS, which is a R2-resistant starch, has been shown to increase carbohydrate-degrading bacteria, such as Bifidobacteria, and decrease bacteria with proteolytic activity, such as *E. coli* [14, 17, 18]. RPS has also been shown to reduce the concentrations of gut microbiome-derived uremic toxins in pigs [19]. While animal and human studies involving resistant starch have shown the ability to change the gut microbiota and reduce the amount of uremic toxins, there are limited studies in patients with CKD [14].

In one study conducted in individuals with CKD already on dialysis, a reduction in uremic toxins following high-amylose cornstarch consumption for 6 weeks was observed [20]. High-amylose cornstarch is ~ 60% resistant starch by dry weight, whereas the RPS to be used in this proposal is ~ 70% resistant starch and ~ 10% other dietary fibres [17]. Here, we will conduct a 2-period randomized double-blind cross-over trial to investigate whether the consumption of RPS as an adjunctive therapy to current standards of CKD care will reduce uremic toxins and symptoms by altering gut microbiota in patients with CKD.

The results of this study will add to the evidence for the efficacy of RPS in patients with CKD and will form the basis of a larger multicentre randomized controlled trial testing the effect of RPS on delaying CKD progression and dialysis initiation.

### Data management

#### Source data

Source documents are where data are first recorded and from which participants’ CRF data are obtained. CRF entries will be considered source data if the CRF is the site of the original recording (e.g. there is no other written or electronic record of data). All documents will be stored safely in confidential conditions. On all trial-specific documents, other than the signed consent forms, master list, and remuneration forms, the participant will be referred to by the trial participant code, not by name.

#### Access to data

Direct access will be granted to authorized representatives from the sponsor, the host institution, and the regulatory authorities to permit trial-related monitoring, audits and inspections.

#### Data recording and record keeping

All trial data will be entered from paper CRF or collected via the University of Manitoba REDCap platform, and other source documents will be entered into this REDCap database. The participants will be identified by a unique trial-specific number and/or code in this REDCap database. The participant’s name and any other identifying information will not be included in the REDCap database, except for the signed informed consent form, remuneration form, and study master list. Participants’ home and email addresses and phone numbers will be collected and linked to the study ID and participant name on a physical/electronic study master list which will be used to coordinate trial activities such as home deliveries of the study materials, remuneration cheques, and communication with participants during the trial. This master list will be stored in a locked cabinet at the CDIC or on a password-protected shared drive/computer at CDIC. Participants’ names and addresses will be shared with the delivery staff or couriers during the delivery process, as well as used for mailing remuneration cheques for the participants who do not pick up in person.

Trial data, without identifying participants’ personal information, will be stored in a secure research environment at the University of Manitoba using REDCap. REDCap is implemented locally by the Data Science platform at the George & Fay Yee Centre for Healthcare Innovation at the University of Manitoba. Study virtual visits will be conducted through the University of Manitoba Microsoft Teams platform, which is an externally hosted cloud-based service. Electronic data with identifying participant personal information, such as name and contact information, will be password-protected and stored in an Excel file on a computer at CDIC. Electronic records of signed consent forms will be stored on REDCap, as well as stored in a password-protected secure computer/shared drive at CDIC or in a locked cabinet. Paper signed consent form will be stored in a locked cabinet in CDIC. Other study logs will be organized in a study binder and kept in CDIC. CDIC is secured 24 h per day and has restricted access.

All trial research records will be kept for 25 years. Paper CRFs and source data will be kept in a locked storage container, apart from any personal identifying information at CDIC. Paper files will be disposed of using the confidential document destruction method at CDIC.

Electronic data will be de-identified and retained for 10 years following the end of the study. Electronic data may also be shared in a de-identified form to academic journals for publication purposes. The data may be stored by the academic journal or other open-access repositories under an open-access policy in which case it may be used by other researchers for further data analysis and research purposes. Paper files will be disposed of using the confidential document destruction method.

#### Criteria for the termination of the trial

The trial will be continued until all recruited participants have reached the end of their follow-up and data have been collected, processed, and cleaned. There are no plans for early termination.

#### Procedure for accounting for missing, unused, and spurious data

The number and proportion of missing values will be documented in the clinical study report. Missing values will not be imputed unless otherwise noted. Analyses will exclude data from participants who have missing values for any variable required for the analysis.

When data are observed to be unusual in a way that cannot be explained or ruled to be in error, analyses may be repeated after excluding the record involved. These additional analyses will be presented as sensitivity analyses.

#### Procedures for reporting any deviation(s) from the original statistical plan

All protocol deviations documented in the clinical trial database will be tabulated (if appropriate) and listed in the clinical study report.

#### Recording of adverse events

Given the nature of the intervention, it is very unlikely that any adverse events will be related to the trial. However, all AEs occurring during the trial that are observed by the investigators or reported by the participant will be recorded on the CRF, whether or not attributed to the trial intervention.

The following information will be recorded: description, date of onset and end date, severity, and assessment of relatedness to trial intervention. Follow-up information should be provided as necessary. In case any adverse event is reported, patients will be offered to be seen in the next available clinic visit or within 1 week, whichever is earlier, and will continue to be followed in the clinic until the AE is resolved.

The severity of events will be assessed on the following scale: 1 = mild, 2 = moderate, and 3 = severe.

AEs considered related to the trial intervention as judged by the qualified investigator will be followed either until resolution or the event is considered stable. In case AEs result in withdrawal from the trial, the patients that are withdrawn due to adverse treatment reaction will also be followed by the CKD clinic until the AEs has resolved.

#### Safety reporting

The study team will report AEs to Health Canada and the relevant department/institution heads and the University of Manitoba REB by using appropriate reporting forms.

### Quality assurance procedures

The trial will be conducted in accordance with the currently approved protocol, GCP, relevant regulations, and standard operating procedures.

Regular monitoring may be performed according to GCP. Data may be evaluated for compliance with the protocol and accuracy in relation to source documents. Following written standard operating procedures, the monitors will verify that the clinical trial is conducted and data are generated, documented, and reported in compliance with the protocol, GCP, and the applicable regulatory requirements.

## Data Availability

The publications arising from this trial will follow the ICMJE recommendations for authorship. The results of this trial will be published in a peer-reviewed publication and may be presented at conferences. The summary results of this study will be uploaded to the trial’s ClinicalTrials.gov registry. De-identified data will be made available to other researchers for the purposes of knowledge synthesis activities upon reasonable request.

## Abbreviations

AE: Adverse event
AR: Adverse reaction
PI: Principal investigator
CRA: Clinical research associate
CRF: Case report form
GCP: Good Clinical Practice
CT: Clinical trials
ICF: Informed consent form
REB: Research Ethics Board
SAE: Serious adverse event
SOP: Standard operating procedure
